# A novel thermostable and halophilic thioredoxin reductase from the Red Sea Atlantis II hot brine pool

**DOI:** 10.1371/journal.pone.0217565

**Published:** 2019-05-31

**Authors:** Elham A. Badiea, Ahmed A. Sayed, Mohamad Maged, Walid M. Fouad, Mahmoud M. Said, Amr Y. Esmat

**Affiliations:** 1 Department of Biochemistry, Faculty of Science, Ain Shams University, Cairo, Egypt; 2 Department of Biology, School of Sciences and Engineering, American University in Cairo, New Cairo, Egypt; 3 Children Cancer Hospital, Cairo, Egypt; 4 Faculty of Biotechnology, October University for Modern Sciences and Arts, 6th October City, Cairo, Egypt; Weizmann Institute of Science, ISRAEL

## Abstract

The highly extreme conditions of the lower convective layer in the Atlantis II (ATII) Deep brine pool of the Red Sea make it an ideal environment for the search for novel enzymes that can function under extreme conditions. In the current study, we isolated a novel sequence of a thioredoxin reductase (TrxR) enzyme from the metagenomic dataset established from the microbial community that resides in the lower convective layer of Atlantis II. The gene was cloned, expressed and characterized for redox activity, halophilicity, and thermal stability. The isolated thioredoxin reductase (ATII-TrxR) was found to belong to the high-molecular-weight class of thioredoxin reductases. A search for conserved domains revealed the presence of an extra domain (Crp) in the enzyme sequence. Characterization studies of ATII-TrxR revealed that the enzyme was halophilic (maintained activity at 4 M NaCl), thermophilic (optimum temperature was 65°C) and thermostable (60% of its activity was retained at 70°C). Additionally, the enzyme utilized NADH in addition to NADPH as an electron donor. In conclusion, a novel thermostable and halophilic thioredoxin reductase has been isolated with a unique sequence that adapts to the harsh conditions of the brine pools making this protein a good candidate for biological research and industrial applications.

## Introduction

The Red Sea is characterized by the presence of large bodies of salty water at its bottom. These hypersaline anoxic deep-sea basins are known as brine pools. Most of these pools are characterized by high temperatures and salinity. Twenty-five brine pools have been found in the Red Sea [1]. The Atlantis II hot-brine deep (ATII) is the largest and best-characterized pool in the Red Sea [2]. This pool is located at a depth of approximately 2200 m near the central rift (21°21' N, 38°04' E) [3], is less than 100 m thick and covers an area of 60 km^2^ [4]. The pool is stratified into different layers, including the brine-seawater interface and the upper, middle and the lower convective layers [2]. The lower convective layer (LCL) is the lowermost layer that presents an exceptional combination of different harsh environmental stressors. This layer is characterized by a high temperature (68°C), extreme salinity (26%), acidic pH (5.3), extremely low levels of light and oxygen, and high concentrations of heavy metals [4–6]. The ATII brine pool, as an extreme environment, is expected to be a source of novel microbial enzymes that can function under harsh conditions, making these enzymes highly suitable for biotechnological and industrial applications [7].

Metagenomic sequencing is the sequencing of all microbial DNA from an environmental niche [8]. This approach enables the characterization of the entire microbiome of a given environment; hence, this method can be used to identify the environmental factors that are responsible for shaping different microbial community structures. Additionally, metagenomics can identify phenotypic changes that result from drastic environmental changes. Therefore, metagenomics is a powerful tool for studying the genomes of a wide range of unculturable microbes and investigating the potentials of these microbes as sources of novel enzymes [7]. The metagenomic studies on the Red Sea brine pools started during the KAUST/WHOI/HCMR oceanographic cruise of the RV *Aegaeo* in March/April 2010 to reveal the microbial communities in these hot pools. Using 454 pyrosequencing technology, a metagenomic dataset for the microbial communities present in the LCL of Atlantis II Deep was established. Using functional screening of the metagenome of the ATII brine pool in the Red Sea, Mohamed *et al*. [9] identified a novel esterase (EstATII), which was thermophilic, halotolerant and resistant to heavy metals. Subsequently, Sayed *et al*. [6] identified a novel mercuric reductase (merA) in a metagenomic database from the ATII brine pool.

Thioredoxin reductase (TrxR) (EC.1.8.1.9) is a flavoprotein enzyme that is widely distributed and catalyses the reduction of the protein thioredoxin in an NADPH-dependent manner. Thioredoxin plays several key roles in maintaining the redox environment of the cell [10]. The thioredoxin system is important in the defence against reactive oxygen species (ROS), acting in cooperation with either Trx peroxidases [11] or glutathione [12] to reduce H_2_O_2_ and recycle many other antioxidant molecules, including lipoamide/lipoic acid [13], dehydroascorbate [14] and alpha-tocopherol quinone. Thioredoxin reductase contains a redox-active disulfide adjacent to the flavin ring. Two classes of thioredoxin reductase with two different modes of catalysis have been reported. The first class has a molecular weight of 35 kDa/subunit and is mainly found in prokaryotes, archaea, and lower eukaryotes, while the other class of the thioredoxin reductases is found in higher eukaryotes and has a molecular weight of 55 kDa/subunit [15]. Reducing equivalents are transferred from the apolar flavin-binding site to the substrate by different mechanisms in the two classes. In the low-molecular-weight thioredoxin reductase, interconversion between two conformations occurs twice in each catalytic cycle. After reduction of the disulfide by the flavin, the pyridine nucleotide domain rotates with respect to the flavin domain to expose the nascent dithiol for reaction with thioredoxin; this motion repositions the pyridine ring adjacent to the flavin ring. In the high-molecular-weight enzyme, an additional redox-active group shuttles the reducing equivalent from the apolar active site to the protein surface. Both classes of TrxR contain an NADPH-binding site and obtain reducing equivalents from NADPH [10].

The thioredoxin system has been isolated from different species, but isolation from an extreme environment with unique conditions, such as the LCL of the ATII Deep brine pool in the Red Sea, has not been conducted. As a part of the metagenomics-based studies of the Red Sea brine pools, this study was conducted to isolate and characterize the thioredoxin reductase enzyme from the deep brine pool of Atlantis II at the LCL of this pool in the Red Sea.

## Materials and methods

Water samples were collected from the LCL of the Atlantis II brine pool in the Red Sea (2200 m below the surface) at 21°20.72’ N and 38°04.59’ E during the KAUST/WHOI/HCMR oceanographic cruise of the RV *Aegaeo* in March/April 2010 as part of the collaboration between King Abdullah University for Science and Technology (KAUST) and Woods Hole Oceanographic Institution (WHOI). All details about the samples and sampling location as well as all necessary permits obtained for the described field studies were described in detail by Siam *et al*. (2). The methods used for sample processing, DNA extraction from microbes trapped in a 0.1-μm filter, measurement of the recovered DNA concentration and DNA pyrosequencing by the GS FLX Titanium Pyrosequencing Kit have been described in detail previously [6, 9].

### Sequence screening against InterPro, the conserved domain database, and BLAST

Open reading frame (ORF) calling was performed using the MetaGene Annotator tool [16]. An operon containing a TrxR ORF was identified by BLAST search [17] against the non-redundant (nr) database. The ORF shared 87% amino acid sequence identity to a putative thioredoxin reductase from *Cupriavidus metallidurans* (accession no. WP011516654.1). The protein families of the ATII-TrxR sequence were determined by screening the sequence against the InterPro protein families database [18]. A conserved domain search was conducted using the conserved domain database (CDD) maintained by NCBI [19].

### Multiple sequence alignment and phylogenetic tree construction

For phylogenetic analysis, sequences of 37 thioredoxin reductases from different species were retrieved from the NCBI protein database. Multiple sequence alignment of the retrieved thioredoxin reductase sequences along with the ATII-TrxR sequence was performed using ClustalX version 2.1 software [20], and a phylogenetic tree was constructed using the neighbour-joining method [21] and MEGA 7 software [22].

### Modelling of the three-dimensional structure of ATII-TrxR

Structure prediction of the isolated thioredoxin reductase enzyme sequence was carried out using I-TASSER protein fold recognition server. I-TASSER simulations generated a large ensemble of structural conformations, called decoys. To select the final models, I-TASSER uses the SPICKER program to cluster all the decoys based on the pair-wise structure similarity. The confidence of each model was quantitatively measured by C-score that was calculated based on the significance of threading template alignments and the convergence parameters of the structure of the assembled simulations [23].

### Identification of the functional regions in ATII-TrxR

Functionally important regions in the ATTII-TrxR protein sequence were identified by submitting the sequence to the ConSurf web server (http://consurf.tau.ac.il/) [24], which estimates the degree of conservation of amino acid sites among close sequence homologs.

### Physicochemical properties of the ATII-TrxR protein sequence

The frequencies of each amino acid and its classification in the ATII-TrxR protein were determined using the BioWord tool [25].

### Prediction of the number of salt bridges and hydrogen bonds

Prediction of the number of salt bridges in the ATII-TrxR protein was performed using ESBRI (Evaluating the Salt BRIdges in proteins) server [26] with default parameters. Similarly, the number of salt bridges was predicted in the corresponding TrxRs from normal and extreme environments. In addition, potential hydrogen bonds were predicted using an online software tool (http://cib.cf.ocha.ac.jp/bitool/HBOND/) [27].

### Expression and purification of ATII-LCL TrxR

The coding sequence of thioredoxin reductase deduced from the LCL metagenomic dataset was amplified, and then, the amplified DNA fragments were cloned into the TOPO TA cloning vector (Invitrogen, USA) and sequenced by the Sanger sequencing method using an ABI 3730XI DNA sequencer (Thermo Fisher Scientific, USA). The sequence was then synthesized (GL Biochem (SHA) Ltd., China) after codon optimization to increase the expression levels of this protein in *Escherichia coli*. The synthesized gene was cloned into the expression vector pET-28a (+) (Novagen, USA). PCR was performed on positive clones using a Veriti thermal cycler (Applied Biosystem, CA, USA) as follows: initial denaturation at 95°C for 5 min; 35 cycles of denaturation at 95°C for 30 seconds, annealing at 51°C for 30 seconds and extension at 72°C for 1.30 min; and final extension at 72°C for 7 min. Plasmids were extracted using the QIAprep Spin Miniprep Kit (Qiagen, Venlo, Netherlands) and then transformed into chemically competent *E*. *coli* BL21 (DE3) cells (Novagen, USA) for protein expression. An overnight culture of *E*. *coli* BL21 (DE3) transformed with pET-28a (+) containing the ATII-LCL TrxR gene was grown in LB broth containing 50 μg/ml kanamycin in a shaking incubator at 37°C and 200 rpm. The culture was diluted 100-fold with fresh LB broth containing 50μg/ml kanamycin (10ml of overnight culture was diluted 100-fold to produce one liter of cell culture). The culture was grown at 37°C to an optical density at 600 nm of ~0.6. Expression was then induced with 0.25 mM isopropyl β-D-1-thiogalactopyranoside (IPTG), followed by further incubation at 25°C and 150 rpm overnight. The cell pellets were collected by centrifugation (1500 ×g for 20 min), exposed to multiple cycles of freezing and thawing, and then re-suspended at 2 ml/g cells in His-binding buffer (20 mM sodium phosphate, 0.5 M NaCl, 20 mM imidazole). The cell suspension was sonicated on ice for three 30-second bursts with 30-second intervals on ice using a Soniprep 150 Plus instrument (MSE, London, UK). The cell lysate was collected after centrifugation at 15000 ×g for 20 min at 4°C. The supernatant was filtered through a filter membrane CN 0.2μm (Thermo Scientific, USA) and then applied to a HisTrap column after equilibration of the column. The protein was eluted from the column using increasing concentrations of imidazole (100, 300 and 500 mM). The protein concentration was determined using the Bicinchoninic acid (BCA) assay kit (Thermo Scientific, USA), from the standard curve using bovine serum albumin as standard. The accuracy of the BCA assay was calculated from the standard curve and found to be 99.44% (R^2^ 0.9944) (S3 Fig).

Protein purification was assessed by 10% SDS-PAGE. The pooled fractions of the eluted protein from the HisTrap purification were dialysed against phosphate-buffered saline using ÄKTA purifier (GE Healthcare, New York, USA) according to the manufacturer instructions. Briefly, the sample loop of the ÄKTA purifier system was filled manually using a syringe with the pooled fractions of ATII-TrxR enzyme, and then connected to the injection valve. The flow path continued through the flow restrictor that generated a constant backpressure to eliminate the risk of air bubbles entering the UV cell. The system was applied to run using UNICORN software by adjusting the flow rate at 1ml/min and a 0.5 ml fractionation volume. During the run, the progress of the method was monitored at UNICORN screen, and the result was captured (S1 Fig). The amount of cofactor FAD bound to the ATII-TrxR protein was estimated form the flavin absorbance at 450 nm using an extinction coefficient of 11.3 mM^-1^ cm^-1^ [28].

### Thioredoxin reductase enzyme assays

#### DTNB reduction assay

To assess the redox activity of the ATII-TrxR enzyme, the activity was spectrophotometrically measured by reduction of 5,5'-dithiobis (2-nitrobenzoic acid) (DTNB) in the presence of NADPH [29]. The final assay reaction (1 ml) contained 100 mM potassium phosphate buffer (pH 7.4), 1 mM EDTA, 2 mM DTNB and 100 nM TrxR. The reaction was initiated with 0.1 mM NADPH, and the increase in absorbance was monitored at 412 nm for 3 min at 25°C. The enzyme activity was calculated as micromoles of NADPH oxidized per minute [30]. The specific activity of the enzyme was calculated by dividing the enzyme activity by the enzyme concentration in the sample. One unit of enzyme activity was defined as the amount of enzyme that produced 2 μmol of 2-nitro-5-thiobenzoate per μmol of NADPH oxidized per minute (ε412 nm = 13.6 mM^−1^ cm^−1^) [31].

#### Insulin reduction assay

The redox activity of ATII-TrxR was also measured by an insulin reduction assay [32]. The final assay reaction (1 ml) contained 100 mM potassium phosphate buffer (pH 7.4), 1 mM EDTA, 0.1 mM human insulin (1 mg/ml), thioredoxin protein (ATII-Trx) in the range of 0.2–4 μM, and ATII-TrxR enzyme. The reaction was initiated by the addition of 0.1 mM NADPH. The enzyme activity was calculated from the decrease in absorbance at 340 nm using a molar extinction coefficient of 6.22 mM^-1^ cm^-1^ [30].

### Biochemical characterization of ATII-TrxR enzyme

#### Effect of NaCl concentration on ATII-TrxR activity (Halophilicity)

The redox activity of ATII-TrxR was assayed by the DTNB reduction assay in the presence of different concentrations of NaCl ranging from 0–4 M.

#### Thermophilicity of the ATII-TrxR enzyme

The optimum temperature for ATII-TrxR enzyme activity was determined by the DTNB reduction assay at increasing temperatures (30–75°C) under standard conditions as described above, and the specific activity at each temperature was calculated.

#### Thermal stability analysis

Forty-microlitre aliquots of the ATII- TrxR enzyme were incubated at a range of temperatures (30–100°C) for 10 min. The tubes were centrifuged at 15,000 ×g for 10 min to remove any precipitated enzyme. The supernatant was assayed for enzyme activity using the DTNB reduction assay as described above. Additionally, the enzyme was incubated for varying periods (0–60 min) at two different elevated temperatures (70 and 90°C), and the residual activity was measured relative to the enzyme activity determined at 30°C.

## Results

### Sequence screening against InterPro and the conserved domain database and BLAST search for the ORF selected in this study

The contig identified in this study, designated as contig00316, was 11637 bp in length. ORF calling by the MetaGeneAnnotator tool identified a 1659-bp ORF encoding a putative thioredoxin reductase (ATII-TrxR) with a sequence identity of 87% with a thioredoxin reductase from *C*. *metallidurans*. The ORF was aligned with nr BLASTX and screened against the CDD and InterPro web interfaces. The results showed that the protein encoded by contig00316_ORF belonged to the pyridine nucleotide-disulfide oxidoreductase class-II family and contained two distinctive domains (a cyclic nucleotide-binding domain and a FAD/NAD (P)-binding domain) (Table 1).

**Table 1:** InterPro, CDD and nr Blastx search results for the open reading frame (ORF) selected in this study.

Database	Contig00316_ORF	
**InterPro**		
Protein family membership	Pyridine nucleotide-disulphide oxidoreductase, class-II (IPR000103)	
Domains and repeats	• aa (13–146) Cyclic nucleotide-binding domain (IPR000595) • aa (222–252) FAD/NAD(P)-binding (IPR023753)	
**CDD Search**		
Name	TrxB	Crp
E-Value	2.41e-55	5.12e-07
Interval	234–522	28–146
Description	Thioredoxin reductase (Posttranslational modification, protein turnover, chaperones)	cAMP-binding domain of CRP or a regulatory subunit of cAMP-dependent protein kinases
Accession number	COG0492	COG0664
**nr BLASTX**		
E-Value	0.0	
Description	thioredoxin reductase [*Cupriavidus metallidurans*]	
% Identity	87%	
Hit Coverage	99%	

### Multiple sequence alignment and phylogenetic tree construction

**Fig 1:**
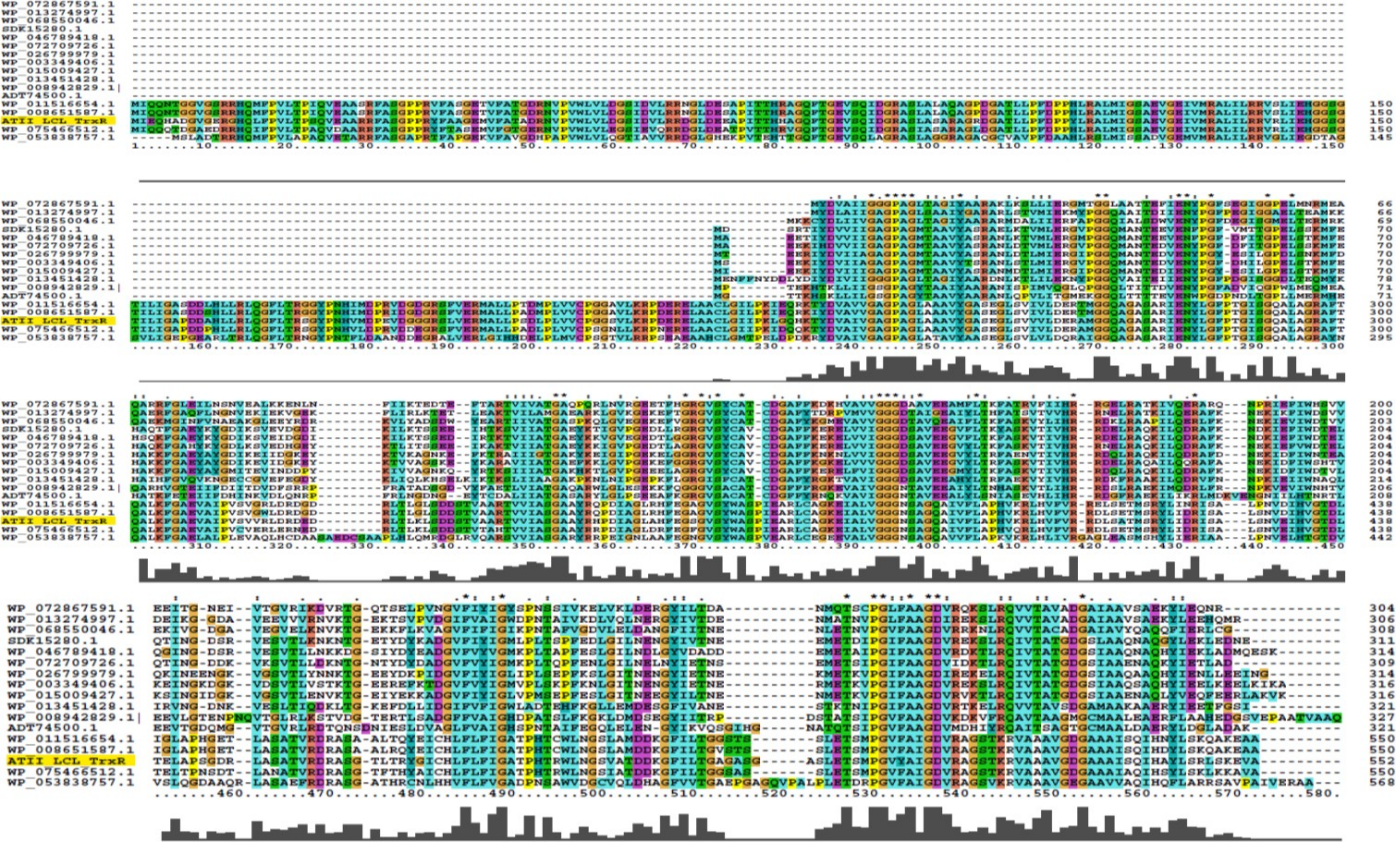
Multiple sequence alignment of ATII-TrxR with thioredoxin reductases from different species. The alignments were carried out using ClustalX version 2.1. Conserved amino acids are coloured. As shown in the figure, the FAD-binding domain is conserved in all the thioredoxin reductases and identifiable by the GXGXXG sequence. Additionally, the NADPH-binding motif was detected in all the sequences (GGGXXA). ATII-TrxR was aligned against 16 different TrxRs from different species and environments: WP_072867591.1 (*Desulfotomaculum thermosubterraneum*), WP_013274997.1 (*Thermosediminibacter oceani*), WP_068550046.1 (*Thermosulfidibacter takaii*), SDK15280.1 (*Jeotgalicoccus halophilus*), WP_046789418.1 (*Salinicoccus halodurans*), WP_072709726.1 (*Salinicoccus alkaliphilus*), WP_026799979.1 (*Pontibacillus halophilus*), WP_003349406.1 (*Bacillus methanolicus*), WP_015009427.1 (*Amphibacillus xylanus*), WP_013451428.1 (*Calditerrivibrio nitroreducens*), WP_008942829.1| (*Oceanibaculum indicum*), ADT74500.1 (*Escherichia coli W*), WP_011516654.1 (*Cupriavidus metallidurans*), WP_008651587.1 (*Cupriavidus* sp. *HMR-1*), WP_075466512.1 (*Ralstonia solanacearum*) and WP_053838757.1 (*Xanthomonas translucens*).

**Fig 2:**
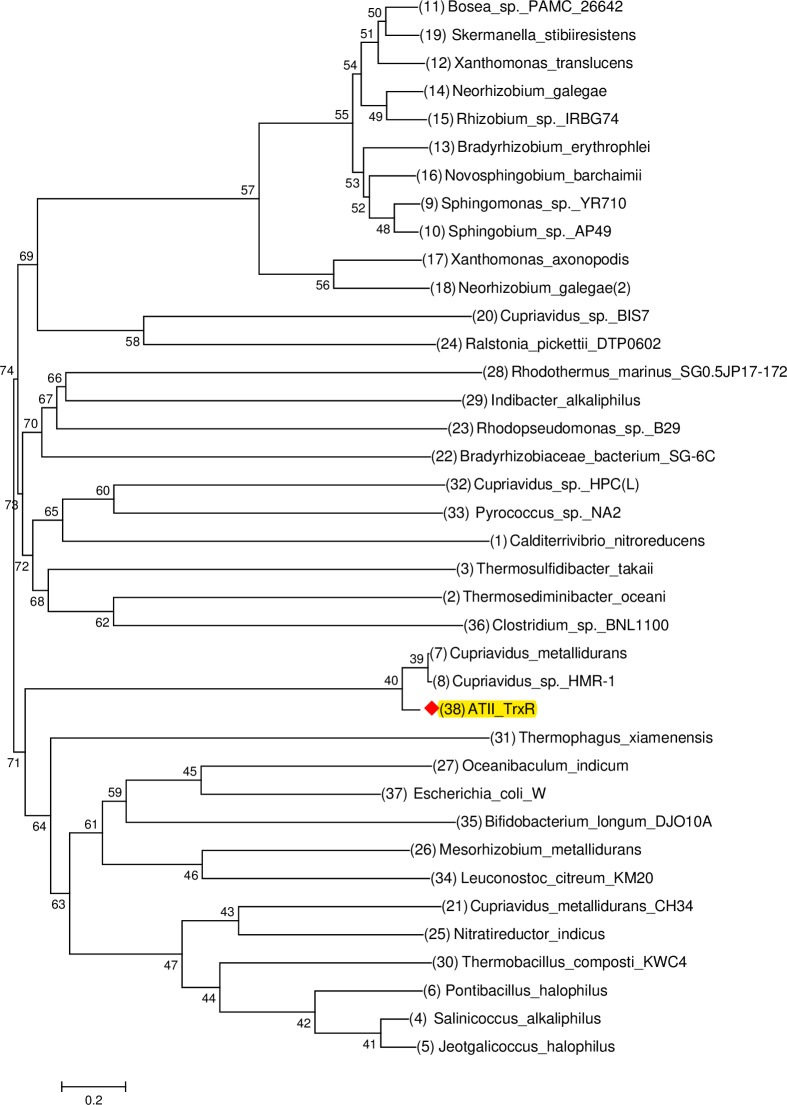
Phylogenetic analysis of ATII-TrxR. The evolutionary history of ATII-TrxR with thioredoxin reductases from different species was inferred using the neighbour-joining method. The tree is drawn to scale, with branch lengths in the same units as those of the evolutionary distances used to infer the phylogenetic tree. The analysis involved 37 amino acid sequences. Evolutionary analyses were conducted in MEGA7. As shown in the figure, the phylogenetic tree of the thioredoxin reductase protein sequence extracted from the LCL of Atlantis II brine pools of the Red Sea revealed that the ATII-TrxR sequence was closely related to the sequences from the heavy metal-resistant bacteria *Cupriavidus metallidurans* and *Cupriavidus* sp. *HMR-1*.

The ATII-TrxR protein sequence was aligned with 16 different sequences of TrxR, including both classes of thioredoxin reductase (low molecular weight and high molecular weight). Alignments showed that ATII-TrxR was classified as a high-molecular-weight TrxR. The conserved FAD-binding motif (GXGXXG) was identified in all the sequences, and the NADPH-binding motif (GGGXXA) was also detected (Fig 1). The neighbour-joining phylogenetic tree showed that ATII-TrxR was closely related to the sequences from the heavy-metal-resistant bacteria *C*. *metallidurans* and *Cupriavidus* sp. *HMR-1* (Fig 2).

### Modelling of the three-dimensional structure of the ATII-TrxR enzyme

**Fig 3:**
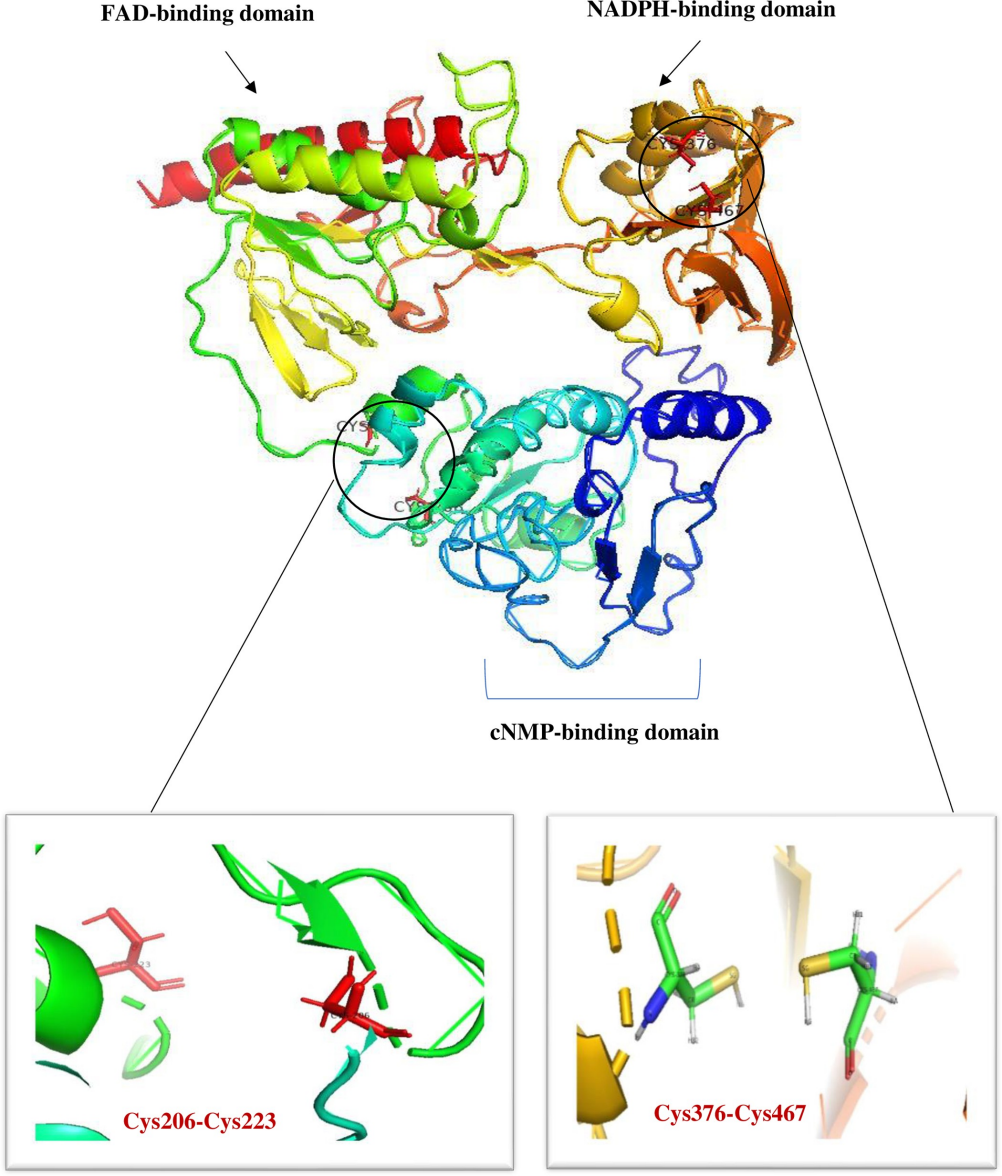
Predicted three-dimensional structural model of ATII-TrxR. The structure is composed of an N-terminal domain (blue) and a C-terminal domain (red). Both the FAD- and NADPH-binding domains are illustrated in the figure, and the additional domain representing the Crp superfamily and its binding domain for cNMP are shown. Also, the cysteine residues involved in the redox centres (Cys206-Cys223) and (Cys376-Cys467) are illustrated.

Prediction of the three-dimensional structure of the ATII-TrxR protein revealed that the ATII-TrxR enzyme was composed of three domains, including the FAD- and NADPH-binding domains, which are identified in all thioredoxin reductases. An additional domain representing the Crp superfamily and the cNMP-binding domain of this superfamily was also identified at the N-terminus of the structure (Fig 3).

### Identification of the functional regions in the ATII-TrxR enzyme

**Fig 4:**
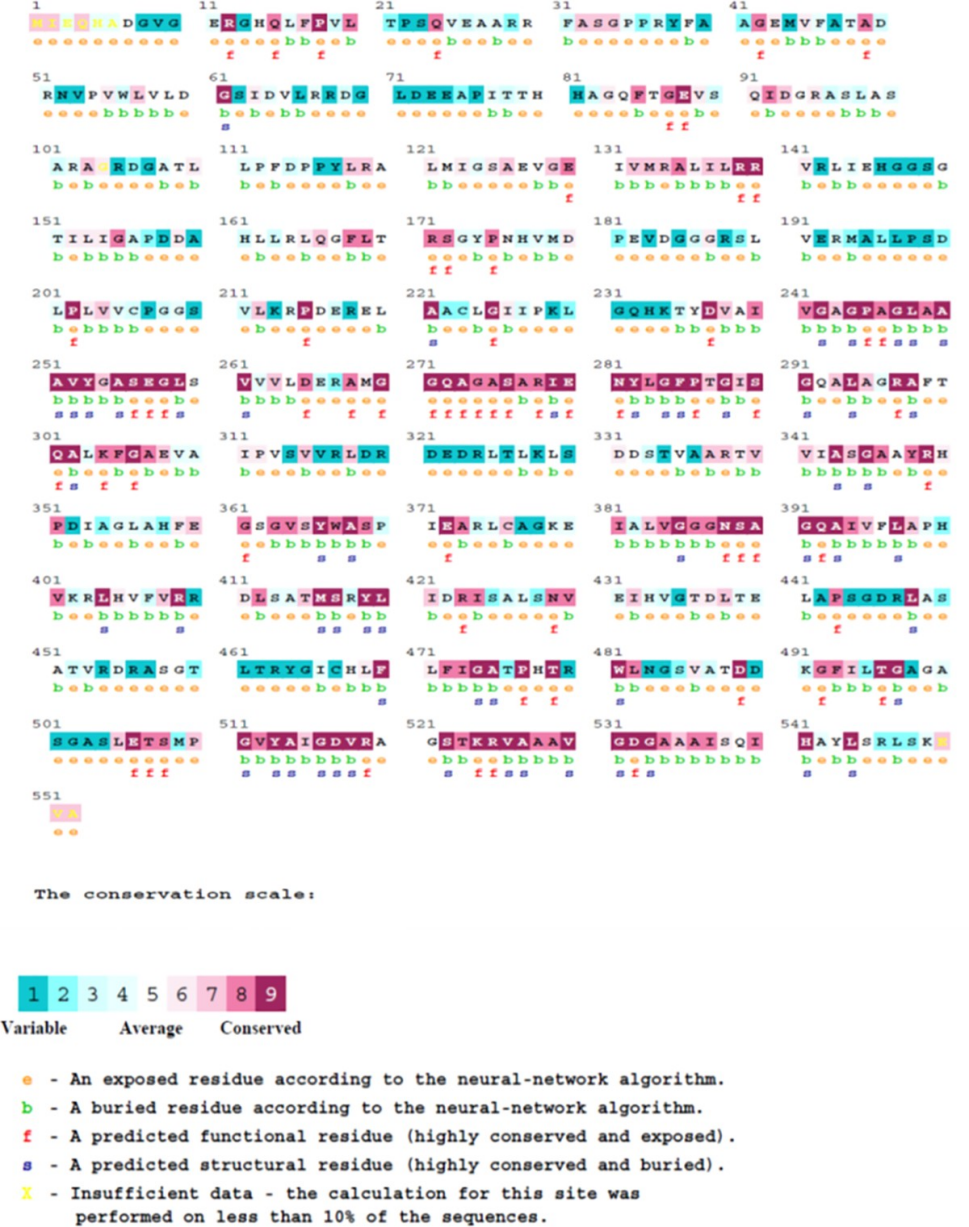
Identification of functional regions in ATII-TrxR enzyme sequence by the ConSurf server. The ConSurf server, based on multiple sequence alignments, predicted that almost all of the glutamic acid (E), arginine (R) and glycine (G) residues are functional residues that are highly conserved and exposed to the outside of the ATII-TrxR protein structure.

The conserved and functional regions in the ATII-TrxR protein sequence were identified by submitting the amino acid sequence to the ConSurf web server (http://consurf.tau.ac.il/). The ConSurf server, based on multiple alignments, predicted that almost all of the glutamic acid (E), arginine (R) and glycine (G) residues were functional residues that were highly conserved and exposed to the outside of ATII-TrxR protein structure (Fig 4). The exposure of these residues might contribute to the formation of salt bridges between negatively and positively charged amino acid residues on the protein surface and could play a major role in protein stability.

### Evaluation of the predicted number of salt bridges and hydrogen bonds in ATII-TrxR

The number of salt bridges and H bonds in ATII-TrxR was compared with those in the corresponding TrxR enzymes from normal (mouse type 2 protein) and harsh (*C*. *metallidurans* and *Cupriavidus* sp. *HMR-1*) environments. The number of salt bridges in the ATII-TrxR protein was predicted using ESBRI server. However, the number of salt bridges in ATII-TrxR (77 salt bridges) was only slightly higher than that in the enzymes isolated from the extreme conditions (70 and 73 salt bridges in *C*. *metallidurans* and *Cupriavidus* sp. *HMR-1*, respectively). The potential number of hydrogen bonds in the ATII-TrxR enzyme was evaluated as one of the factors that might contribute to protein stability under extreme conditions. Additionally, the predicted number of hydrogen bonds in ATII-TrxR was found to be 373, which was higher than that of thioredoxin reductases from the best hit bacteria, *C*. *metallidurans* and *Cupriavidus* sp. *HMR-1* (353 and 351 hydrogen bonds, respectively), as well as the mouse type 2 protein (340 hydrogen bonds) (Table 2).

**Table 2:** Predicted number of salt bridges and H-bonds in ATII-TrxR enzyme and the corresponding enzyme templates.

Protein sequence	Number of salt bridges	Number of H-bonds
ATII-TrxR	77	373
TrxR (*Cupriavidus metallidurans)*	70	353
TrxR *(Cupriavidus HMR-1*)	73	351
TrxR (Mouse Type 2)	21	340

### Physicochemical properties of the ATII-TrxR protein sequence

**Fig 5:**
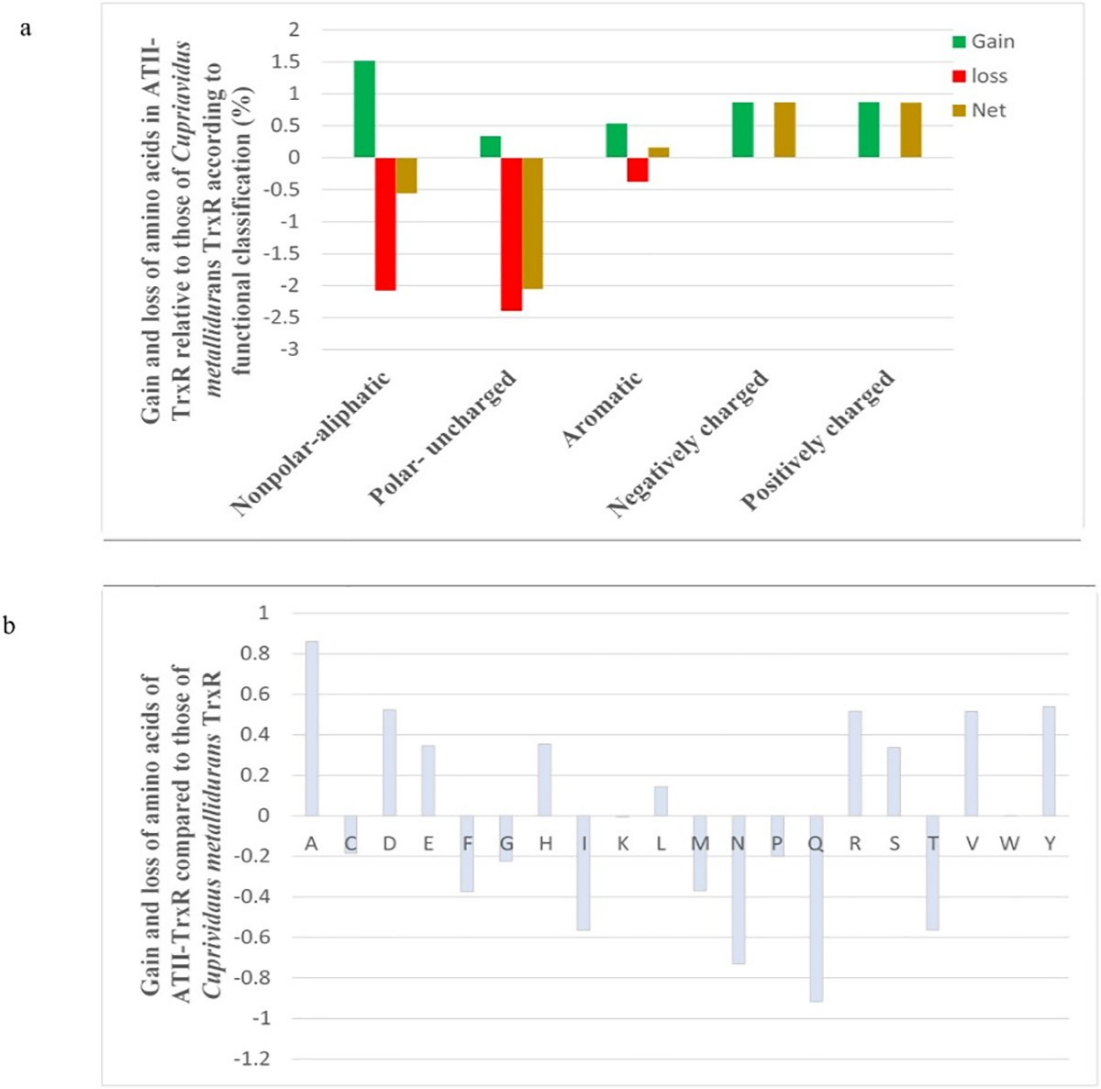
**a) Distribution percentages of different classes of amino acids in ATII-TrxR relative to that of *Cupriavidus metallidurans*. b) Pattern of amino acid substitutions in ATII-TrxR relative to those of *Cupriavidus metallidurans*.** a) The amino acid compositions of TrxR from ATII-LCL and *Cupriavidus metallidurans* were compared, and the histograms show the net change in the number of each of the listed amino acids in the ATII-LCL enzyme. b) Frequency of substitutions in ATII-LCL plotted against the corresponding residue in the *Cupriavidus metallidurans* TrxR enzyme.

The amino acid sequence of ATII-TrxR was analysed and compared to that of the bacterial TrxR from the top hit *C*. *metallidurans*. The ATII-TrxR sequence was found to contain a higher percentage of negatively and positively charged amino acids than the TrxR sequence of *C*. *metallidurans* by 0.87 and 0.865%, respectively (Fig 5A). This finding indicated that the higher potential number of salt bridges in the ATII-TrxR sequence than in the sequence from the metal-resistant bacterium *C*. *metallidurans* was due to the increased number of charged amino acids, which led to the formation of salt bridges between the negatively and positively charged amino acids. These results were confirmed by analysing the frequency of substitutions of each amino acid in the ATII-TrxR enzyme compared to the protein sequence of TrxR from *C*. *metallidurans* (Fig 5B). The results showed that alanine, threonine, glutamic acid, aspartic acid, arginine and histidine residues were more abundant in ATII-TrxR than in the protein from *C*. *metallidurans*, while the opposite was true for glutamine and asparagine.

### Expression and purification of ATII-TrxR

**Fig 6:**
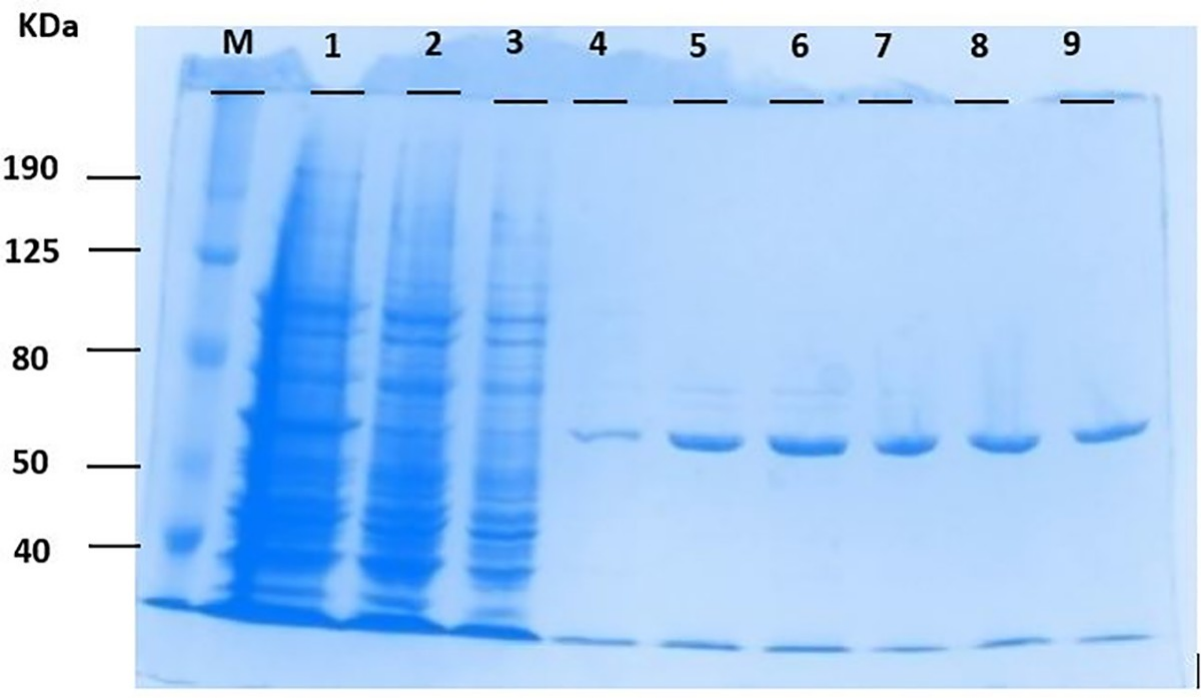
SDS-PAGE of the recombinant ATII-TrxR protein. Aliquots from the HisTag purification process of the ATII-TrxR protein from total bacterial proteins were analysed by 10% SDS-PAGE as described in the experimental procedures. M: molecular weight marker; lane 1: induced sample along with all bacterial proteins; lanes 2 & 3: bacterial flow-through-1 & flow-through-2; lanes 4–9: different fractions of eluted and purified ATII-TrxR protein with a size of 57.8 kDa.

After expression and purification of the recombinant ATII-TrxR protein, different samples of purified ATII-TrxR protein were taken for SDS-PAGE. All *E*. *coli* proteins (lanes 2 and 3) were washed out, while eluted and purified fractions of the recombinant ATII-TrxR protein were retained (lanes 4–9). The purified protein had a molecular weight of 57.8 kDa (Fig 6). All purified fractions were then collected and dialyzed by an ÄKTA purifier to eliminate and separate co-purified proteins from the previous step for biochemical assays. The binding of ATII-TrxR protein to the cofactor FAD was confirmed by the presence of two coinciding peaks at their corresponding wavelengths 280 and 450 nm, respectively (S1 Fig). The fraction of FAD bound to the protein was found to be 1.87 mol/mol of native enzyme.

### Redox activity, Michaelis-Menten plots and kinetics parameters of ATII-TrxR

The catalytic activity of ATII-TrxR demonstrated that the enzyme could reduce the substrate thioredoxin (isolated from the same environment at Atlantis II Deep), with K_m_ and k_cat_ values of 1.03 μM and 28.4 S^-1^, respectively (Table 3). Additionally, the enzyme reduced DTNB (model substrate for TrxR enzymes) with K_m_ and k_cat_ values of 98.32 μM and 5.63 S^-1^, respectively. Furthermore, ATII-TrxR showed high specificity towards NADPH and NADH as cofactors with K_m_ values of 5.21 μM and 4.17 μM, respectively, and k_cat_ values of 8.96 S^-1^ and 1.695 S^-1^, respectively (Table 3). All kinetic parameters of the enzyme were calculated by fitting experimental data (non-linear regression) to the Michaelis-Menten equation using GraphPad prism 8 (GraphPad, La Jolla, CA, USA).

**Table 3:** Kinetic parameters of ATII-TrxR with different substrates.

Substrate	K_m_ (μM)	k_cat_ (S^-1^)	k_cat_/ K_m_ (M^-1^ S^-1^)
NADPH ^a^	5.21±1.1	8.96±1.8	1.7x10^6^
NADH ^a^	4.17 ±2.3	1.695±0.98	0.41 x10^6^
DTNB ^a^	98.32±3.5	5.63±0.95	5.7x10^4^
ATII-Trx ^b^	1.03±0.07	28.4±3.1	27.57x10^6^

Kinetic constants for recombinant ATII-TrxR were determined in assays performed at 25°C and 2M NaCl concentration. Results are means ± SD of triplicate experiments.

^a^ Measured using DTNB reduction assay as described in Materials.

^b^ Measured using insulin reduction assay as described in Materials.

### Characterization of the ATII-TrxR enzyme

#### Halophilicity of the ATII-TrxR enzyme

**Fig 7:**
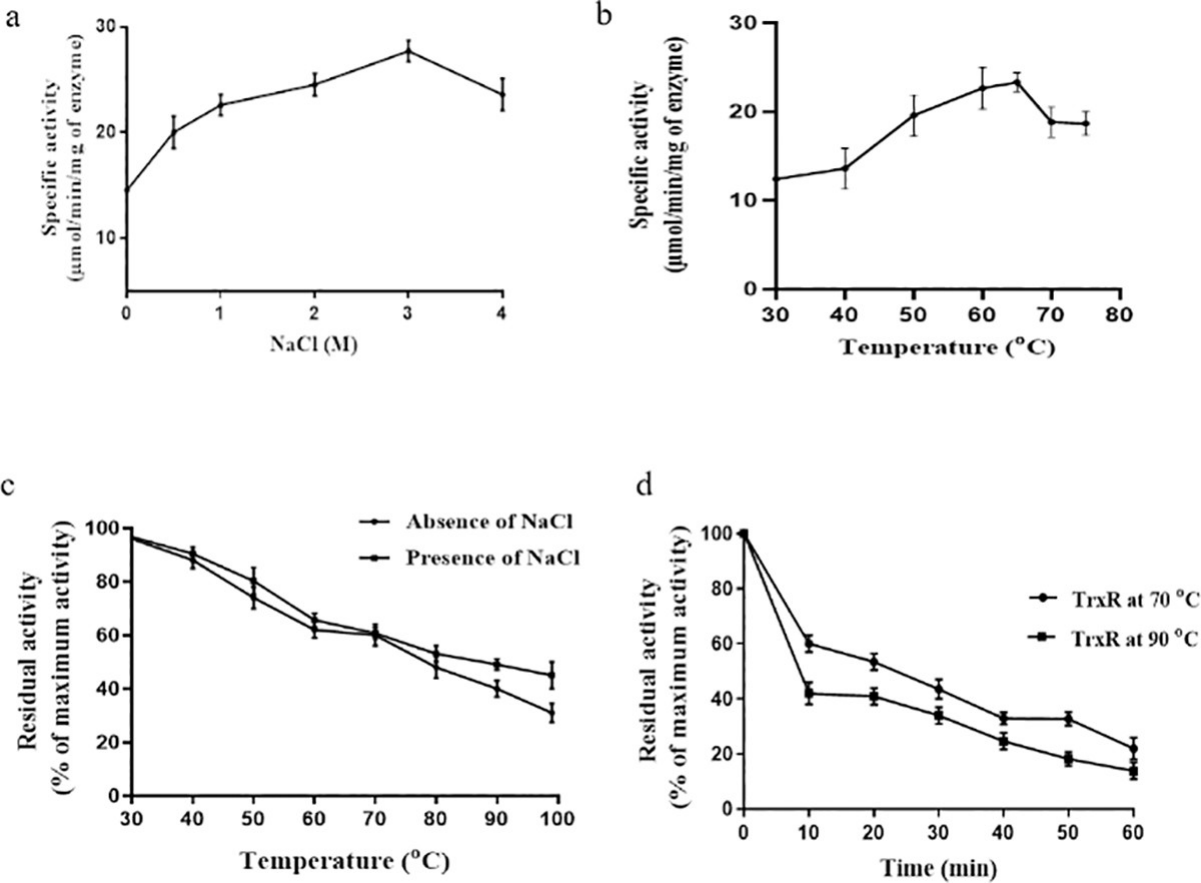
Characterization of the ATII-TrxR enzyme. **a**) Redox activity of the ATII-TrxR enzyme at different molar concentrations of NaCl (halophilicity). **b)** Effect of increasing temperature on ATII-TrxR enzyme activity. The figure shows that the enzyme is thermophilic with an optimum temperature of 65°C. **c)** Residual activity of ATII-TrxR at different temperatures in the absence and presence of 2 M NaCl. **d)** Thermal stability of ATII-TrxR as a function of time.

The effects of different NaCl concentrations (0–4 M) on the redox activity of the ATII-TrxR enzyme were determined using a DTNB reduction assay. ATII-TrxR enzyme activity was found to increase with increasing concentrations of NaCl, reaching a maximum activity of 27.71 μmol/min/mg at 3 M NaCl; then, the activity decreased slightly, reaching 23.58 μmol/min/mg at 4 M (Fig 7A).

#### Thermophilicity of the ATII-TrxR enzyme

The thermophilicity of the ATII-TrxR enzyme was determined by measuring the reductive enzyme activity at increasing temperatures (30, 40, 50, 60, 65, 70, 75°C); the enzyme showed maximum activity at 65°C (Fig 7B).

#### Thermal stability of the ATII-TrxR enzyme

The effect of increasing temperature on ATII-TrxR enzyme activity was assayed by incubating TrxR at increasing temperatures (30 to 100°C) for 10 min, after which, the residual activity was measured by the DTNB reduction assay. ATII-TrxR showed marked thermal stability; the enzyme retained 60% of its activity after incubation at 70°C for 10 min, but the activity was markedly reduced to 39% following incubation at 99°C for 10 min (Fig 7C). Additionally, the thermal stability of ATII-TrxR was studied by incubating the enzyme at two temperatures higher than the environmental temperature (70 and 90°C) for different durations until enzyme activity was lost (0–60 min). The ATII-TrxR enzyme retained 43 and 33% of its activity after incubation for 30 min at 70 and 90°C, respectively. Upon increasing the duration of the thermal treatment, the enzyme started to lose most of its activity, exhibiting 20 and 13% of its maximum activity after incubation for 1 h at 70 and 90°C, respectively (Fig 7D).

## Discussion

Functional metagenomics is a powerful experimental approach for studying gene function in uncultivated microbial communities [33]. This function-based approach involves isolation of environmental DNA directly from microbial communities and examination of the functions of the corresponding encoded proteins. This approach has allowed the discovery of novel enzymes for which functional prediction could not be conducted based only on DNA sequence [34]. Using such an approach, we isolated a gene encoding a novel thioredoxin reductase enzyme from a water sample taken from the LCL of the Red Sea Atlantis II brine pool. Thioredoxin reductase (TrxR) plays an important role in maintaining the redox balance and counteracting oxidative stress inside cells [35].

The isolated ATII-TrxR gene consists of 1659 bp, corresponding to 552 amino acids, with a protein molecular weight of 57.8 kDa (Fig 6). This finding indicates that the enzyme belongs to the high-molecular-weight TrxR class. Two superfamilies were identified by a domain search: the pyridine nucleotide-disulfide superfamily (Pfam ID: 07992), which is a characteristic domain of the thioredoxin reductase enzyme, and the Crp superfamily (Pfam ID: 00027), which includes the cNMP-binding domain (Table 1). Phylogenetic analysis of the protein sequence showed that the isolated ATII-TrxR was closely related to thioredoxin reductases from *C*. *metallidurans* and *Cupriavidus* sp. *HMR-1*, which are heavy-metal-resistant bacteria that are normally found in industrial sediments and wastes that contain high heavy metal concentrations (Fig 2 and Table 1). Sequence alignment of the ATII-TrxR protein revealed 87% identity with a sequence from the top hit *C*. *metallidurans*, a gram-negative non-spore forming motile, heavy metal-resistant bacterium [36, 37]. This bacterium contains a group of genes that are abundant in the genome and encode heavy metal exporters and potential metal-binding proteins that play a major role in cell detoxification [38].

The purified ATII-TrxR is a dimeric protein that has a typical FAD-binding motif (GXGXXG) near the N-terminus and an NADPH-binding motif (GGGXXA) near the middle of the protein, which is common in all enzymes with thioredoxin reductase activity (Fig 1). The amino acid sequence of ATII-TrxR differs from that of the top hit *C*. *metallidurans* by 13% (Table 1). The major differences are represented by the acidic and basic amino acids, showing polar-uncharged amino acid substitution, which might contribute to the formation of salt bridges between amino acids with opposite charges, thus increasing the protein stability (Fig 5A). The kinetic parameters of ATII-TrxR were investigated using different substrates. DTNB is a commonly used substrate for thioredoxin reductases because the disulfide bond in this compound is highly reactive, providing a convenient method for studying enzymatic reduction [39]. The K_m_ of ATII-TrxR enzyme for DTNB is reported to be 98.32 μM (Table 3), demonstrating the ability of the enzyme to catalyse direct reduction of DTNB, which is a common feature of H-TrxRs and is not normally observed in L-TrxRs [35]. This value is similar to that of the TrxR from *Babesia microti*, which exhibited K_m_ of 135.15 μM [40], but is much lower than the K_m_ values reported for cytosolic and mitochondrial H-TrxRs from rat liver (660 and 530 μM, respectively) [41] and *Plasmodium falciparum* (465 μM) [42], as well as the K_m_ value for lower eukaryotes (*Aspergillus nidulans*, 5.1 mM) [43]. In addition, the K_m_ value of ATII-TrxR for NADPH was found to be 5.21 μM, which is very similar to the values for other H-TrxRs, including the cytosolic TrxR from rat liver (6 μM), *Drosophila melanogaster* (6.5 μM), *Giardia duodenalis* (8 μM) [12, 30, 44] and *Aspergillus nidulans* (3.7 μM) [43]. On the other hand, the K_m_ of ATII-TrxR is lower than that of TrxR from *Deinococcus radiophilus* (12.5 μM) [45]. The enzyme has also been shown to utilize NADH as an electron donor, with K_m_ and k_cat_ values of 4.17 μM and 1.695 S^-1^, respectively. Furthermore, the purified ATII-TrxR enzyme can reduce the corresponding substrate, thioredoxin (ATII-Trx) isolated from the same environment at Atlantis II deep [46], exhibiting K_m_ value of 1.03 μM (Table 3). This value is lower than the K_m_ values of thioredoxin reductases from *Aspergillus nidulans* and *S*. *Cerevisiae* towards their homologous thioredoxins (3.4 μM and 3.45 μM, respectively) [43, 47].

The catalytic efficiency, i.e., the k_cat_/K_m_ ratio, is a useful index for comparison of the relative rates of an enzyme that acts on alternative or competing substrates [48]. Comparison of the kinetic parameters of the ATII-TrxR enzyme towards the different substrates reveals that the enzyme exhibits much greater efficiency towards its substrate thioredoxin (this protein was also identified and isolated from the metagenomic datasets for the LCL of Atlantis II Deep, and biochemical analysis of this protein showed marked halophilicity and thermal stability), represented by k_cat_ of 28.4 S^-1^ and k_cat_/K_m_ of 27.57×10^6^ M^-1^ S^-1^, followed by NADPH and NADH as cofactors and DTNB as a substrate (k_cat_ of 5.63 S^-1^ and k_cat_/ K_m_ of 5.7×10^4^ M^-1^ S^-1^).

The predicted number of salt bridges and hydrogen bonds in ATII-TrxR was assessed as a preliminary indicator of thermal stability. The ATII-TrxR protein contains an unusual number of salt bridges in the predicted structural model. Salt bridges are electrostatic interactions between acidic and basic amino acid side chains [49] and contribute to the protein thermal stability [50]. Usually, thermostable proteins show an over-representation of salt bridges and benefit from the electrostatic stabilization conferred by salt bridges [49]. The number of salt bridges in ATII-TrxR was found to be 4 times higher than that in the mouse type 2 TrxR from the normal environment [51]. Halophilicity studies have demonstrated that ATII-TrxR can tolerate high concentrations of salt up to 4 M NaCl (Fig 7A). To the best of our knowledge, many thioredoxin reductase genes have been computationally identified from halophiles, especially archaea, and the sequences of these genes have been submitted to the database, but information regarding the biochemical characteristics of these genes or the tolerance of these genes to high salt concentrations could not be found.

Halophilic microorganisms such as those found in the Atlantis II brine pool require more than 2.5 M salt for optimal growth [52]. These organisms have adapted to the elevated salinity in their surrounding medium via two mechanisms, namely, the salt-in and the salt-out approaches [53]. In the salt-out approach, the organism uses ion pumps to extrude considerable amounts of inorganic solutes while maintaining the intracellular osmotic pressure by accumulation of organic solutes. The most commonly used organic osmolytes are glycerol, betaine, ectoine and small amino acids, such as glycine and histidine [54]. The salt-in mechanism involves the accumulation of molar concentrations of KCl in the cytoplasm [55]. Proteins adapted to high salt concentrations often exhibit a predominance of acidic residues on their surfaces [56]. This observation is confirmed by the conservation scale of the ATII-TrxR enzyme sequence, which revealed that most of the glutamic acid residues are functional and exposed to the surface (Fig 4 and S2 Fig), and these residues in turn contribute to enzyme stability and function. Glutamic acid has a strong ability to bind water, thus enabling protein function and adaptation to extreme conditions by maintaining a proper hydration shell [57].

The ATII-TrxR enzyme is a thermophilic enzyme, i.e., the enzyme activity increases with temperature, with maximum activity observed at 65°C (optimum temperature of the ATII-TrxR enzyme). ATII-TrxR exhibited remarkable thermal stability, retaining 60% of its maximum activity after 10 min of incubation at 70°C in the absence and presence of NaCl (Fig 7C). The enzyme retained 40% of its activity at 90°C in the absence of salt and 49% in the presence of NaCl at the same temperature, and the enzymatic activity was markedly reduced at 99°C. It is clear that the presence of salt does not affect the residual activity of the enzyme after thermal treatment. Additionally, the enzyme was shown to tolerate temperatures higher than that at the LCL of Atlantis II Deep (65°C), exhibiting residual activity of 43 and 33% after incubation for 30 min at 70°C and 90°C, respectively (Fig 7D). Because ATII-TrxR contains a large number of salt bridges, these bridges might contribute to the stability and adaptation of the enzyme to high temperatures. Many thioredoxin reductase enzymes were identified from thermophilic and hyperthermophilic microorganisms. The TrxR isolated from *Thermotoga maritima* showed high thermophilicity, with the activity of this enzyme increasing with the increasing temperatures up to 95°C, retaining 60% of its activity after incubation at 80°C for 28 h [58] In contrast, in terms of catalysis, the ATII-TrxR enzyme has a higher catalytic efficiency for NADPH as a cofactor (K_m_ = 5.21 μM) (Table 3) than the TrxR of *T*. *maritima* (K_m_ = 780 μM). Another thermophilic thioredoxin reductase, isolated from the archaeon *Pyrococcus horikoshii*, exhibited no decrease in enzyme activity after thermal treatment for 1 h at 100°C, which is the optimum temperature for the growth of *P*. *horikoshii* [59]. Notably, although the previously described thioredoxin reductases isolated from thermophilic and hyper-thermophilic microorganisms exhibited higher optimum temperatures and thermal stability than ATII-TrxR isolated from the hot brine pool at Atlantis II Deep, these enzymes showed no activity at high salt concentrations up to 4 M NaCl. Thus, the ATII-TrxR enzyme has dual properties maintaining activity under high temperature and at high salt concentrations. Thermophilic and thermotolerant enzymes are of great economic importance because they are often highly resistant to denaturation and stable at elevated temperatures making them a good candidate for biotechnological and industrial applications compared to mesophilic enzymes [60].

In conclusion, we identified a novel thioredoxin reductase sequence from the LCL of Atlantis II Deep (ATII-TrxR) brine pools in the Red Sea. The sequence contains an extra domain, namely Crp domain at the N-terminus. The ATII-TrxR enzyme is halophilic and thermostable (retaining 60% of its activity at 70°C). Analysis of the biochemical properties and sequence highlighted the adaptation of this protein to the extreme temperature and salinity of the hot brine pool from which the enzyme is originated.

ATII-TrxR doesn’t contain selenocysteine in its amino acid sequence, but due to its unique properties of halophilicity and thermal stability this enzyme can be furtherly tagged with selenocysteine motif (C-terminal tetrapeptide motif: -Gly-Cys-Sec-Gly-COOH, known as a sel-tag) according to the method provided by Cheng *et al*. [61]. The selenocysteine has different properties from cysteine due to its lower pKa value and stronger nucleophilic effect, which can be used in many selenium-dependent applications, including residue-specific radiolabeling with gamma or positron emitters, improved phasing in X-ray crystallography, introduction of ^77^Se for NMR spectroscopy, and the analysis or tailoring of enzymatic reactions involving thiol or redox selenolate chemistry [62, 63].

## Supporting information

S1 Fig Dialysis of purified fractions of ATII-TrxR by ÄKTA purifier system. (TIF)

S2 Fig Ribbon diagram of ATII-TrxR enzyme structure showing the predominance of acidic residues (glutamic acid). (TIF)

S3 Fig Standard curve of bovine serum albumin using BCA assay showing accuracy of 99.44%. (TIF)

## References

[1] Wang Y, Li JT, He LS, Yang B, Gao ZM, Cao HL, et al Zonation of Microbial Communities by a Hydrothermal Mound in the Atlantis II Deep (the Red Sea). PloS one. 2015;10(10):e0140766 Epub 2015/10/21. 10.1371/journal.pone.0140766 26485717 PMC4613831

[2] Siam R, Mustafa GA, Sharaf H, Moustafa A, Ramadan AR, Antunes A, et al Unique prokaryotic consortia in geochemically distinct sediments from Red Sea Atlantis II and discovery deep brine pools. PloS one. 2012;7(8):e42872 Epub 2012/08/24. 10.1371/journal.pone.0042872 22916172 PMC3423430

[3] Kamanda Ngugi D, Blom J, Alam I, Rashid M, Ba-Alawi W, Zhang G, et al Comparative genomics reveals adaptations of a halotolerant thaumarchaeon in the interfaces of brine pools in the Red Sea. ISME J. 2015;9(2):396–411. Epub 2014/08/12. 10.1038/ismej.2014.137 25105904 PMC4303633

[4] Laurila TE, Hannington MD, Leybourne M, Petersen S, Devey CW, Garbe-Schönberg D. New insights into the mineralogy of the Atlantis II Deep metalliferous sediments, Red Sea. Geochemistry, Geophysics, Geosystems. 2015;16(12):4449–78. 10.1002/2015GC006010

[5] Adel M, Elbehery AH, Aziz SK, Aziz RK, Grossart HP, Siam R. Viruses-to-mobile genetic elements skew in the deep Atlantis II brine pool sediments. Scientific reports. 2016;6:32704 Epub 2016/09/07. 10.1038/srep32704 27596223 PMC5011723

[6] Sayed A, Ghazy MA, Ferreira AJ, Setubal JC, Chambergo FS, Ouf A, et al A novel mercuric reductase from the unique deep brine environment of Atlantis II in the Red Sea. The Journal of biological chemistry. 2014;289(3):1675–87. 10.1074/jbc.M113.493429 24280218 PMC3894346

[7] Behzad H, Ibarra MA, Mineta K, Gojobori T. Metagenomic studies of the Red Sea. Gene. 2016;576(2 Pt 1):717–23. Epub 2015/11/04. 10.1016/j.gene.2015.10.034 . 26526132

[8] Mick E, Sorek R. High-resolution metagenomics. Nature Biotechnology. 2014;32:750 10.1038/nbt.2962 25101744

[9] Mohamed YM, Ghazy MA, Sayed A, Ouf A, El-Dorry H, Siam R. Isolation and characterization of a heavy metal-resistant, thermophilic esterase from a Red Sea brine pool. Scientific reports. 2013;3:3358 Epub 2013/11/29. 10.1038/srep03358 . 24285146 PMC6506439

[10] Williams CH, Arscott LD, Muller S, Lennon BW, Ludwig ML, Wang PF, et al Thioredoxin reductase two modes of catalysis have evolved. European journal of biochemistry. 2000;267(20):6110–7. Epub 2000/09/30. . 11012662 10.1046/j.1432-1327.2000.01702.x

[11] Cha MK, Kim IH. Thioredoxin-linked peroxidase from human red blood cell: evidence for the existence of thioredoxin and thioredoxin reductase in human red blood cell. Biochemical and biophysical research communications. 1995;217(3):900–7. Epub 1995/12/26. 10.1006/bbrc.1995.2856 . 8554614

[12] Kanzok SM, Fechner A, Bauer H, Ulschmid JK, Muller HM, Botella-Munoz J, et al Substitution of the thioredoxin system for glutathione reductase in Drosophila melanogaster. Science (New York, NY). 2001;291(5504):643–6. Epub 2001/02/07. 10.1126/science.291.5504.643 . 11158675

[13] Arner ES, Nordberg J, Holmgren A. Efficient reduction of lipoamide and lipoic acid by mammalian thioredoxin reductase. Biochemical and biophysical research communications. 1996;225(1):268–74. Epub 1996/08/05. 10.1006/bbrc.1996.1165 . 8769129

[14] May JM, Mendiratta S, Hill KE, Burk RF. Reduction of dehydroascorbate to ascorbate by the selenoenzyme thioredoxin reductase. The Journal of biological chemistry. 1997;272(36):22607–10. Epub 1997/09/05. 10.1074/jbc.272.36.22607 . 9278416

[15] Saccoccia F, Angelucci F, Boumis G, Carotti D, Desiato G, Miele AE, et al Thioredoxin reductase and its inhibitors. Current protein & peptide science. 2014;15(6):621–46. Epub 2014/05/31. 10.2174/1389203715666140530091910 24875642 PMC4275836

[16] Noguchi H, Taniguchi T, Itoh T. MetaGeneAnnotator: detecting species-specific patterns of ribosomal binding site for precise gene prediction in anonymous prokaryotic and phage genomes. DNA Research: An International Journal for Rapid Publication of Reports on Genes and Genomes. 2008;15(6):387–96. Epub 2008/10/23. 10.1093/dnares/dsn027 18940874 PMC2608843

[17] Altschul SF, Gish W, Miller W, Myers EW, Lipman DJ. Basic local alignment search tool. Journal of molecular biology. 1990;215(3):403–10. Epub 1990/10/05. 10.1016/S0022-2836(05)80360-2 . 2231712

[18] Mitchell A, Chang HY, Daugherty L, Fraser M, Hunter S, Lopez R, et al The InterPro protein families database: the classification resource after 15 years. Nucleic acids research. 2015;43(Database issue):D213–21. Epub 2014/11/28. 10.1093/nar/gku1243 25428371 PMC4383996

[19] Marchler-Bauer A, Derbyshire MK, Gonzales NR, Lu S, Chitsaz F, Geer LY, et al CDD: NCBI's conserved domain database. Nucleic acids research. 2015;43(Database issue):D222–6. Epub 2014/11/22. 10.1093/nar/gku1221 25414356 PMC4383992

[20] Thompson JD, Higgins DG, Gibson TJ. CLUSTAL W: improving the sensitivity of progressive multiple sequence alignment through sequence weighting, position-specific gap penalties and weight matrix choice. Nucleic acids research. 1994;22(22):4673–80. Epub 1994/11/11. 10.1093/nar/22.22.4673 7984417 PMC308517

[21] Nei NSaM. The Neighbor-joining Method: A New Method for Reconstructing Phylogenetic Trees. Molecular Biology and Evolution 1987;4:406–25. 10.1093/oxfordjournals.molbev.a040454 3447015

[22] Kumar S, Stecher G, Tamura K. MEGA7: Molecular Evolutionary Genetics Analysis Version 7.0 for Bigger Datasets. Mol Biol Evol. 2016;33(7):1870–4. Epub 2016/03/24. 10.1093/molbev/msw054 . 27004904 PMC8210823

[23] Yang J, Yan R, Roy A, Xu D, Poisson J, Zhang Y. The I-TASSER Suite: protein structure and function prediction. Nature methods. 2015;12(1):7–8. Epub 2014/12/31. 10.1038/nmeth.3213 25549265 PMC4428668

[24] Glaser F, Pupko T, Paz I, Bell RE, Bechor-Shental D, Martz E, et al ConSurf: Identification of Functional Regions in Proteins by Surface-Mapping of Phylogenetic Information. Bioinformatics. 2003;19(1):163–4. 10.1093/bioinformatics/19.1.163 12499312

[25] Anzaldi LJ, Muñoz-Fernández D, Erill I. BioWord: A sequence manipulation suite for Microsoft Word. BMC Bioinformatics. 2012;13(1):124 10.1186/1471-2105-13-124 22676326 PMC3546851

[26] Costantini S, Colonna G, Facchiano AM. ESBRI: A web server for evaluating salt bridges in proteins. Bioinformation. 2008;3(3):137–8. 19238252 10.6026/97320630003137 PMC2639689

[27] Baker EN, Hubbard RE. Hydrogen bonding in globular proteins. Progress in Biophysics and Molecular Biology. 1984;44(2):97–179. 10.1016/0079-6107(84)90007-5. 6385134

[28] Whitby LG. A new method for preparing flavin-adenine dinucleotide. Biochemical Journal. 1953;54(3):437–42. 10.1042/bj0540437 13058921 PMC1269010

[29] Holmgren A, Bjornstedt M. Thioredoxin and thioredoxin reductase. Methods in enzymology. 1995;252:199–208. Epub 1995/01/01. . 7476354 10.1016/0076-6879(95)52023-6

[30] Luthman M, Holmgren A. Rat liver thioredoxin and thioredoxin reductase: purification and characterization. Biochemistry. 1982;21(26):6628–33. Epub 1982/12/21. . 7159551 10.1021/bi00269a003

[31] Cohen G, Yanko M, Mislovati M, Argaman A, Schreiber R, Av-Gay Y, et al Thioredoxin-thioredoxin reductase system of Streptomyces clavuligerus: sequences, expression, and organization of the genes. Journal of bacteriology. 1993;175(16):5159–67. Epub 1993/08/01. 10.1128/jb.175.16.5159-5167.1993 8349555 PMC204983

[32] Arner ES, Zhong L, Holmgren A. Preparation and assay of mammalian thioredoxin and thioredoxin reductase. Methods in enzymology. 1999;300:226–39. Epub 1999/01/27. . 9919525 10.1016/s0076-6879(99)00129-9

[33] Lam KN, Cheng J, Engel K, Neufeld JD, Charles TC. Current and future resources for functional metagenomics. Frontiers in Microbiology. 2015;6(1196). 10.3389/fmicb.2015.01196 26579102 PMC4625089

[34] Ufarté L, Potocki-Veronese G, Laville É. Discovery of new protein families and functions: new challenges in functional metagenomics for biotechnologies and microbial ecology. Frontiers in Microbiology. 2015;6:563 10.3389/fmicb.2015.00563 PMC4456863. 26097471 PMC4456863

[35] Hirt RP, Muller S, Embley TM, Coombs GH. The diversity and evolution of thioredoxin reductase: new perspectives. Trends in parasitology. 2002;18(7):302–8. Epub 2002/10/17. . 12379950 10.1016/s1471-4922(02)02293-6

[36] Reith F, Rogers SL, McPhail DC, Webb D. Biomineralization of gold: biofilms on bacterioform gold. Science (New York, NY). 2006;313(5784):233–6. Epub 2006/07/15. 10.1126/science.1125878 . 16840703

[37] Reith F, Fairbrother L, Nolze G, Wilhelmi O, Clode PL, Gregg A, et al Nanoparticle factories: Biofilms hold the key to gold dispersion and nugget formation. Geology. 2010;38(9):843–6. 10.1130/G31052.1

[38] Reith F, Etschmann B, Grosse C, Moors H, Benotmane MA, Monsieurs P, et al Mechanisms of gold biomineralization in the bacterium Cupriavidus metallidurans. Proceedings of the National Academy of Sciences of the United States of America. 2009;106(42):17757–62. Epub 2009/10/10. 10.1073/pnas.0904583106 19815503 PMC2764933

[39] Lacey BM, Hondal RJ. Characterization of Mitochondrial Thioredoxin Reductase from C. elegans. Biochemical and biophysical research communications. 2006;346(3):629–36. 10.1016/j.bbrc.2006.05.095 PMC3687220. 16780799 PMC3687220

[40] Zhao S, Gong H, Zhou Y, Zhang H, Cao J, Zhou J. Identification of a thioredoxin reductase from Babesia microti during mammalian infection. Parasitology research. 2016;115(8):3219–27. Epub 2016/05/12. 10.1007/s00436-016-5084-4 . 27164832

[41] Watabe S, Makino Y, Ogawa K, Hiroi T, Yamamoto Y, Takahashi SY. Mitochondrial thioredoxin reductase in bovine adrenal cortex its purification, properties, nucleotide/amino acid sequences, and identification of selenocysteine. European journal of biochemistry. 1999;264(1):74–84. Epub 1999/08/14. . 10447675 10.1046/j.1432-1327.1999.00578.x

[42] Kanzok SM, Schirmer RH, Turbachova I, Iozef R, Becker K. The thioredoxin system of the malaria parasite Plasmodium falciparum. Glutathione reduction revisited. The Journal of biological chemistry. 2000;275(51):40180–6. Epub 2000/10/03. 10.1074/jbc.M007633200 . 11013257

[43] Thon M, Al-Abdallah Q, Hortschansky P, Brakhage AA. The thioredoxin system of the filamentous fungus Aspergillus nidulans: impact on development and oxidative stress response. The Journal of biological chemistry. 2007;282(37):27259–69. Epub 2007/07/17. 10.1074/jbc.M704298200 . 17631497

[44] Brown DM, Upcroft JA, Upcroft P. A thioredoxin reductase-class of disulphide reductase in the protozoan parasite Giardia duodenalis. Molecular and Biochemical Parasitology. 1996;83(2):211–20. 10.1016/S0166-6851(96)02776-4. 9027754

[45] Seo HJ, Lee YN. Characterization of Deinococcus radiophilus thioredoxin reductase active with both NADH and NADPH. Journal of microbiology (Seoul, Korea). 2010;48(5):637–43. Epub 2010/11/04. 10.1007/s12275-010-0283-7 . 21046342

[46] Gamal, M M. (2014). Towards the Characterization of a Novel Thermohalophilic Antioxidant Thioredoxin from the Metagenome of the Red Sea; LCL of Atlantis II Brine Pool (Master's thesis), AUC, Cairo, Egypt. Retrived from http://dar.aucegypt.edu/handle/10526/3931.

[47] Pedrajas JR, Kosmidou E, Miranda-Vizuete A, Gustafsson JA, Wright AP, Spyrou G. Identification and functional characterization of a novel mitochondrial thioredoxin system in Saccharomyces cerevisiae. The Journal of biological chemistry. 1999;274(10):6366–73. Epub 1999/02/26. 10.1074/jbc.274.10.6366 . 10037727

[48] Eisenthal R, Danson MJ, Hough DW. Catalytic efficiency and kcat/KM: a useful comparator? Trends in biotechnology. 2007;25(6):247–9. Epub 2007/04/17. 10.1016/j.tibtech.2007.03.010 . 17433847

[49] Bosshard HR, Marti DN, Jelesarov I. Protein stabilization by salt bridges: concepts, experimental approaches and clarification of some misunderstandings. Journal of molecular recognition: JMR. 2004;17(1):1–16. Epub 2004/02/12. 10.1002/jmr.657 . 14872533

[50] Lee CW, Wang HJ, Hwang JK, Tseng CP. Protein thermal stability enhancement by designing salt bridges: a combined computational and experimental study. PloS one. 2014;9(11):e112751 Epub 2014/11/14. 10.1371/journal.pone.0112751 25393107 PMC4231051

[51] Eckenroth BE, Rould MA, Hondal RJ, Everse SJ. Structural and biochemical studies reveal differences in the catalytic mechanisms of mammalian and Drosophila melanogaster thioredoxin reductases. Biochemistry. 2007;46(16):4694–705. Epub 2007/03/28. 10.1021/bi602394p 17385893 PMC3687216

[52] Madern D, Ebel C, Zaccai G. Halophilic adaptation of enzymes. Extremophiles: life under extreme conditions. 2000;4(2):91–8. Epub 2000/05/11. . 10805563 10.1007/s007920050142

[53] Andrei AS, Banciu HL, Oren A. Living with salt: metabolic and phylogenetic diversity of archaea inhabiting saline ecosystems. FEMS microbiology letters. 2012;330(1):1–9. Epub 2012/02/22. 10.1111/j.1574-6968.2012.02526.x . 22339687

[54] Siglioccolo A, Paiardini A, Piscitelli M, Pascarella S. Structural adaptation of extreme halophilic proteins through decrease of conserved hydrophobic contact surface. BMC structural biology. 2011;11:50 Epub 2011/12/24. 10.1186/1472-6807-11-50 22192175 PMC3293032

[55] DasSarma S, DasSarma P. Halophiles and their enzymes: negativity put to good use. Current opinion in microbiology. 2015;25:120–6. Epub 2015/06/13. 10.1016/j.mib.2015.05.009 26066288 PMC4729366

[56] Paul S, Bag SK, Das S, Harvill ET, Dutta C. Molecular signature of hypersaline adaptation: insights from genome and proteome composition of halophilic prokaryotes. Genome biology. 2008;9(4):R70 Epub 2008/04/10. 10.1186/gb-2008-9-4-r70 18397532 PMC2643941

[57] Oren A. Adaptation of Halophilic Archaea to Life at High Salt Concentrations In: Läuchli A, Lüttge U, editors. Salinity: Environment—Plants—Molecules. Dordrecht: Springer Netherlands; 2002 p. 81–96.

[58] Yang X, Ma K. Characterization of a Thioredoxin-Thioredoxin Reductase System from the Hyperthermophilic Bacterium Thermotoga maritima. Journal of bacteriology. 2010;192(5):1370–6. 10.1128/JB.01035-09 20061476 PMC2820846

[59] Kashima Y, Ishikawa K. A hyperthermostable novel protein-disulfide oxidoreductase is reduced by thioredoxin reductase from hyperthermophilic archaeon Pyrococcus horikoshii. Archives of biochemistry and biophysics. 2003;418(2):179–85. Epub 2003/10/03. . 14522589 10.1016/j.abb.2003.08.002

[60] Gomes E, de Souza AR, Orjuela GL, Da Silva R, de Oliveira TB, Rodrigues A. Applications and Benefits of Thermophilic Microorganisms and Their Enzymes for Industrial Biotechnology In: Schmoll M, Dattenböck C, editors. Gene Expression Systems in Fungi: Advancements and Applications. Cham: Springer International Publishing; 2016 p. 459–92.

[61] Cheng Q, Stone-Elander S, Arner ES. Tagging recombinant proteins with a Sel-tag for purification, labeling with electrophilic compounds or radiolabeling with 11C. Nature protocols. 2006;1(2):604–13. Epub 2007/04/05. 10.1038/nprot.2006.87 . 17406287

[62] Johansson L, Chen C, Thorell JO, Fredriksson A, Stone-Elander S, Gafvelin G, et al Exploiting the 21st amino acid-purifying and labeling proteins by selenolate targeting. Nature methods. 2004;1(1):61–6. Epub 2005/03/23. 10.1038/nmeth707 . 15782154

[63] Johansson L, Gafvelin G, Arner ES. Selenocysteine in proteins-properties and biotechnological use. Biochimica et biophysica acta. 2005;1726(1):1–13. Epub 2005/06/22. 10.1016/j.bbagen.2005.05.010 . 15967579

