# Supplementary figures and images for: A novel thermostable and halophilic thioredoxin reductase from the Red Sea Atlantis II hot brine pool
Source: PLoS One. 2019 May 31;14(5):e0217565. doi: 10.1371/journal.pone.0217565 (PMC6544261; doi:10.1371/journal.pone.0217565)

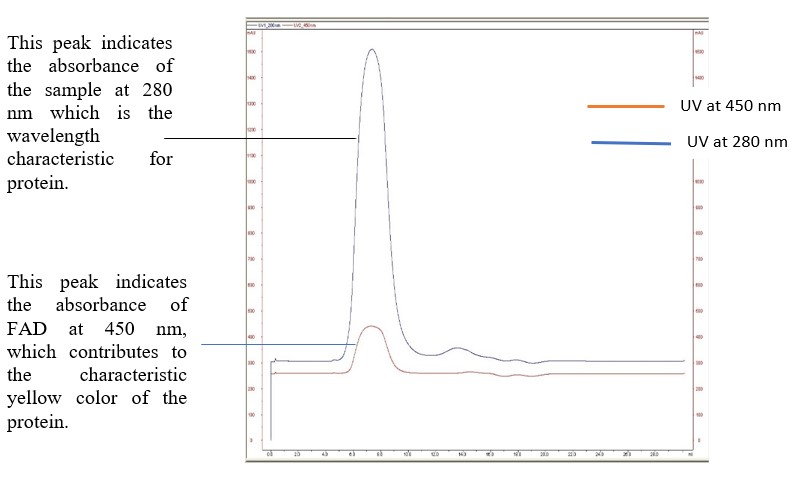

Supplement: S1 Fig — (TIF) [file pone.0217565.s001.tif]

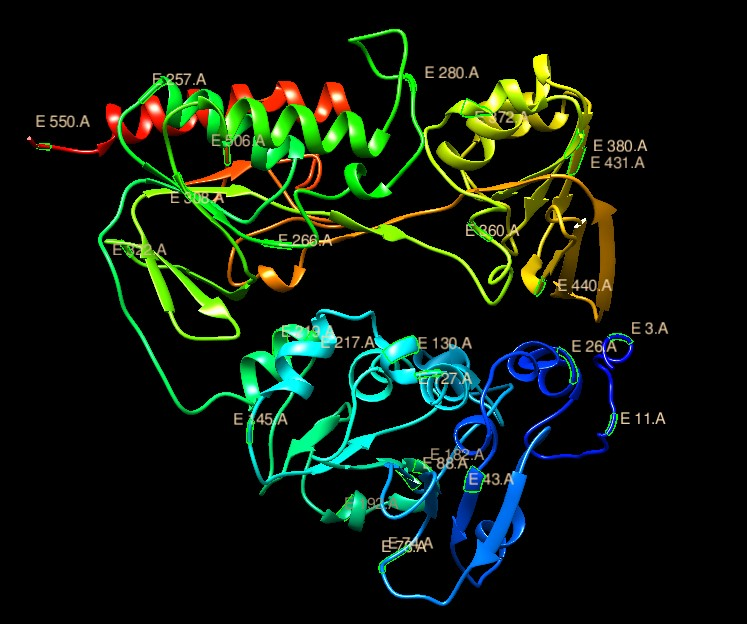

Supplement: S2 Fig — (TIF) [file pone.0217565.s002.tif]

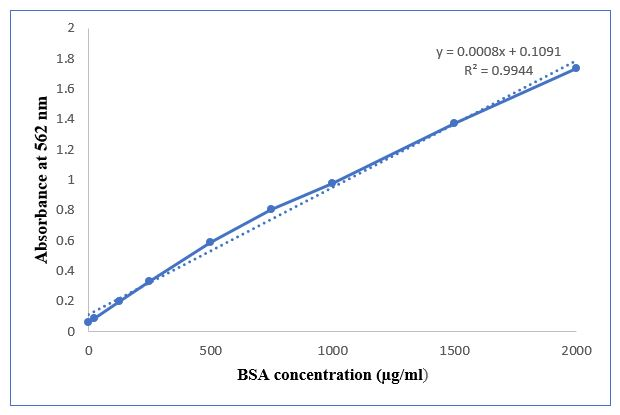

Supplement: S3 Fig — (TIF) [file pone.0217565.s003.tif]
